# Limited Role for *C. pneumoniae*, CMV and HSV-1 in Cerebral Large and Small Vessel Atherosclerosis

**DOI:** 10.2174/1874205X00802010039

**Published:** 2008-07-25

**Authors:** M Voorend, A.J.A.M van der Ven, B Kubat, J Lodder, C.A Bruggeman

**Affiliations:** a Department of Neurology, Cardiovascular research institute Maastricht (CARIM) University Hospital Maastricht; b Department of Medical Microbiology, Cardiovascular research institute Maastricht (CARIM) University Hospital Maastricht; c Department of Internal Medicine, University Hospital Nijmegen; d Department of Pathology, Netherlands Forensic Institute, Rijswijk, The Netherland

**Keywords:** *Chlamydia pneumoniae*, Cytomegalovirus, Herpes Simplex Virus type 1, small vessel disease, brain.

## Abstract

*Aims:* To explore whether* Chlamydia pneumoniae*, Cytomegalovirus and Herpes Simplex Virus type 1 could be detected in large and small cerebral arteries, as well as in an area of brain parenchyma where white matter lesions (leukoaraiosis) can be found, in patients with clinically unmanifested cerebrovascular atherosclerosis. M*ethods and results*( Arterial specimens from the basilar artery and middle cerebral artery, and brain samples from the basal ganglia and periventricular white matter were obtained. Neuropathological changes were assessed in Haematoxylin-Eosin stained sections. Polymerase chain reaction (PCR) was performed on paraffin embedded sections. Subsequently, we performed immunohistochemical staining on samples, which were found positive in PCR. We failed to detect *C. pneumoniae*, CMV, or HSV-1, in any of the cerebral large vessels. In the brain tissue, we found only one case positive for CMV, and one for *C. pneumoniae*. *Conclusions* (our findings suggest a limited role for *C. pneumoniae*, CMV and HSV-1 in cerebral large and small vessel atherosclerosis.

## INTRODUCTION

Atherosclerosis is an inflammatory disease. The ‘response to injury’ hypothesis proposes endothelial dysfunction as the initiating factor, followed by influx of monocyte-derived macrophages and T-lymphocytes [1]. Over the past decade there has been much speculation about the role of microorganisms in the activation and maintenance of this inflammatory response. The microorganisms most extensively investigated in this context are *Chlamydia pneumoniae* (*C. pneumoniae*) and Cytomegalovirus (CMV) [2-4]. Some reports have described the role of Herpes Simplex Virus type 1 (HSV-1) as well [5, 6]. For both *C. pneumoniae* and CMV, the relationship between infection and atherosclerosis was first established in the late 80’s [7, 8]. Two studies showed that patients suffering from cardiovascular disease more often had antibodies against the microorganisms than a matched control group. Pathological studies have shown the presence of *C. pneumoniae* [9], CMV [10] and HSV-1 [11, 12], in arterial specimens from various sites. One study showed the presence of all three microorganisms in the same carotid arterectomy sample [13]. Only a few studies report on the relationship between infection with these microorganisms and cerebrovascular disease (CVD) [14-17]. Even less attention has been paid to the actual presence of these microorganisms in the large cerebral vessels, with only a few reports on the presence of *C. pneumoniae* in these vessels [18-20]. In the study by Rassu *et al. *[19], the presence of CMV was studied as well but the presence of HSV-1 has never been investigated in this context.

Factors involved in CVD may differ from those in cardiovascular disease. CVD may broadly be divided into two entities: large vessel disease (LVD) and small vessel disease (SVD). LVD, which is due to atherosclerosis of the larger cerebral vessels, causes mainly larger, usually called territorial infarcts, which often involve the cortex. The risk factors for large vessel disease are similar to those of cardiovascular disease in general. SVD causes small infarcts, less than 15 mm in diameter on cerebral imaging, which are located in the deep brain structures (basal ganglia and internal capsule) or the pons; these are often referred to as lacunar infarcts. SVD is also the cause of more diffuse damage to the white matter of the brain: leukoaraiosis. The clinical manifestation of either LVD or SVD is consistent over time [21]. Therefore, it is rational to investigate potential causes or factors that sustain these two types of cerebrovascular diseases separately. The role of *C. pneumoniae*, CMV and HSV-1 in SVD has never been investigated in this respect.

The aim of this study was to investigate whether the presence of *C. pneumoniae*, CMV and HSV-1 could be established in the large and small cerebral arteries, as well as in brain parenchyma where leukoaraiosis can be found, in cases with pathological evidence of cerebrovascular atherosclerosis.

## MATERIALS AND METHODS

### Autopsy specimens

Included in the study were autopsy specimens from ten men and nine women, ranging from 33 to 80 years of age (median 70). Patient characteristics are summarized in Table **1**. At autopsy the brain was removed, and immersed in 10% buffered formaldehyde for at least two weeks. The large cerebral vessels were dissected after which the brain was cut in frontal sections. The material was embedded in paraffin and used for routine neuropathological examination.

For this study, specimens were obtained from the areas supplied by small vessels, i.e. the basal ganglia and the periventricular white matter. Arterial specimens included the distal 3-cm of the basilar artery (BA) and the proximal 3 cm of the middle cerebral artery (MCA), directly after it branches from the internal carotid artery. Arterial sites showing macroscopic signs of atherosclerosis were also included. The large vessels were embedded longitudinally.

Paraffin embedded material was used for Haematoxylin/Eosin (HE) staining, immunohistochemistry (IHC) staining and polymerase chain reaction (PCR). Ten 5 μm sections were cut, followed by a 50 μm section. This was serially continued until all material was used. The first of each 5 μm section was used for HE staining; the following 5 μm section was used for IHC. The 50 μm sections were used for PCR; three 50 μm sections were pooled in one serum tube and stored at 4 (C until further processing

### Serology

Post-mortem serum samples were taken from each case. The sera were kept at –20 (C until further processing. *C. pneumoniae* serology was performed using a micro immuno fluorescence test (Labsystems, Helsinki, Finland). Samples were considered positive when titres were 1:32 or higher.

CMV and HSV-1 antibodies were aimed to detect using commercially available Enzyme Linked Immunoabsorbent Assays (ELISA) used in routine diagnosis of these antibodies (Serion, Germany). Samples were considered positive when titres were > 30.

### Tissue Preparation and PCR Analysis

Tissue preparation and DNA extraction was performed using the technique described by Gass *et al*. [22] Prior to DNA isolation, the sections were deparaffinised by adding 700 μl of xylol. After this they were vibrated for 1 minute using the Vortex® and centrifuged for ten minutes at 40000 rotations per minute (rpm) in a Micro 22R( rotator. This procedure was repeated. After deparaffination, the sections were rehydrated by adding 70% ethanol. Again, the samples were vibrated using the Vortex® for 1 minute and centrifuged for ten minutes at 40000 rpm in the Micro 22R( rotator. This also was done twice. Following deparaffination, DNA was extracted from brain tissue with the Wizard Genomic DNA purification kit (Promega, Germany). Then, the purified DNA was subjected to PCR aiming to detect either *C. pneumoniae*, CMV or HSV-1 DNA.

All PCR(s were performed in a total volume of 50 (l, containing 1 μg purified DNA, 0.1 mM of each dNTP, 0.5 μM of each primer:

- *C. pneumoniae*: CPC, 5’-TTA TTA ATT GAT GGT ACA ATA-3’; antisense CPD, 5’-ATC TAC GGC AGT AGT ATA GTT-3’;
- CMV: sense 5’ GCG GGA GAT GTG GAT GGC TTG TAT TAA GGA 3’-antisense 5’-GCA GAC TCT CAG AGG ATC GGC CCC C-3’;
- HSV-1: sense 5’-GCA TCG TCG AGG TGG AC-3’; antisense 5’-CCT GCC ACT TGG TCA TG-3’ DNA

Amplification was carried out in a Perkin Elmer 9600 thermal cycler, using the following cycling conditions:

- *C. pneumoniae*: 37 ^o ^C for ten minutes and 95 ^o ^C for fifteen minutes. 20 cycles of “Touchdown PCR” were performed from 60 ^o ^C to 50 ^o ^C, followed by 40 cycles of 1 minute at 94 ^o^C, 50 ^o^C, and 72 ^o^C.
- CMV: 37 ^o ^C for ten minutes and 95 ^o ^C for fifteen minutes. PCR was performed in 50 cycles of 30 seconds at 95 ^o^C, 70 ^o^C and 72 ^o^C.
- HSV-1: 37 ^o ^C for ten minutes and 95 ^o ^C for fifteen minutes. PCR was performed in 50 cycles of 30 seconds at 94 ^o^C, 69 ^o^C and 72 ^o^C.

After this the PCR products were kept overnight at 15 ^o^C. The PCR products were resolved in a 2% agarose gel, stained with ethidium bromide, and photographed.

### Histology and Immunohistochemistry

Five μm paraffin sections were used for HE staining, performed using standard procedures. The morphological changes were evaluated on routinely processed 5 μm HE sections. The evaluation of the vascular changes was performed by the neuropathologist (BK), who was blinded for the case characteristics. Cerebral large vessel atherosclerosis was graded as mild (+) moderate (++), and severe (+++) when intimal fibrosis, fibro-atheromatous plaques, or fibro-atheromatous plaques with calcification were present, respectively. The changes in the small cerebral vessels were recorded asmild (+), moderate (++), or severe (+++) in eachHE stained block from the basal ganglia or periventricular white matter. Mild SVD was defined as concentric vessel wallthickening in small arteries and arterioles but with mild or minimal luminal narrowing, moderate SVD implied significant luminalnarrowing, but with the lumen spanning more than half the total externaldiameter. In severe SVD the internal vessel diameterwas less than half of the external diameter [23]. Apart from this the presence of lacunar infarcts was recorded.

Since the PCR is the most sensitive technique to detect the presence of microorganisms, we first used the PCR to detect CMV, *C. pneumoniae* and HSV-1. In cases that were positive by PCR, IHC was performed, to identify the specific cells infected with either microorganism. For *C. pneumoniae*, the RR402 mouse monoclonal antibody (against a major outer membrane protein MOMP of *C. pneumoniae*) (DAKO, Denmark) was used as primary antibody (dilution 1:50). This antibody is specific for **C. pneumoniae**[24]. Normal mouse ascites was used as a negative control. Five μm thick paraffin embedded sections were pre-incubated with 2% Bovine Serum Antigen (BSA) in PBS to block non specific staining, after which the slides were incubated with the primary antibody or normal mouse ascites at 37 °C, for one hour, each slide containing two samples, one for the antibody and one for the control, respectively. After washing in Phosphate Buffered Saline (PBS), the sections were incubated with the secondary antibody: biotinylated goat anti-mouse serum (DAKO, Denmark). Immunostaining was performed by the avidin-biotin alkalic phosphatase method (DAKO, Denmark). Fast red was used as a chromogen, after which the sections were counterstained with haematoxylin. Infected Hep-2 cells were used as a positive control; mock-infected Hep-2 cells were used as negative control. The same method was used for the immunohistochemical staining of CMV using an antibody against the E13 gene of human CMV as the primary antibody (Argene Biosoft, France) (dilution1: 50).

## RESULTS

The results are summarized in Table **1**.

### Cerebral Large Vessels

From all cases, either the BA or MCA was available for assessment of cerebral large vessel atherosclerosis. Both vessels could be examined in 14/19 cases. In 5 cases cerebral vessels other then the BA or MCA showed macroscopic signs of atherosclerosis; these were three vertebral artery samples, one anterior cerebral artery, and the intracranial part of one internal carotid artery, respectively. In all but one case microscopic atherosclerotic changes were seen in at least one of the arteries. The case without atherosclerotic changes was a 51-year old male who had died from hepatic encephalopathy. In total, 43 arterial specimens from 19 patients could be investigated. Four of these samples (9%) were without atherosclerotic changes. In the remaining 91% atherosclerotic changes varied from intima fibrosis (35%), and fibro-atheromatous plaques (35%), to fibro-atheromatous plaques with calcification (21%). Territorial cerebral infarction as a result of large vessel atherosclerosis was seen in two cases. More severe atherosclerosis was observed in cases that had died from vascular causes, but this difference was not significant. Using the PCR method, we failed to establish the presence of *C. pneumoniae*, CMV, or HSV-1 in any of the cerebral large vessels. No immunohistochemical staining was done on the cerebral large vessel specimens, since we only performed IHC on specimens positive in PCR.

### Cerebral Small Vessels, Basal Ganglia, Periventricular White Matter

**Fig. (1):**
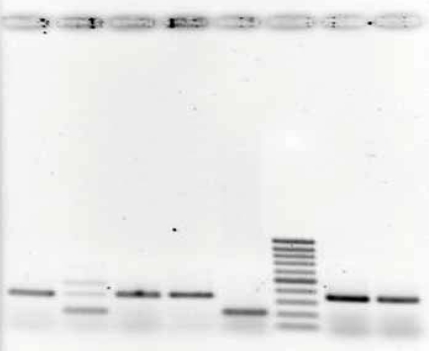
*C. pneumoniae* PCR of periventricular whiter matter. Lane 2 shows sample positive for *C. pneumoniae* at the 207 bp site (case 15), Lanes 1 and 3 are negative samples (cases 13 and 14), lane 4: negative control, lane 5: positive control (*C. pneumoniae* infected Hep-2 cells), lane 6: base pair ladder, lane 7 and 8: test efficiency controls

**Fig. (2):**
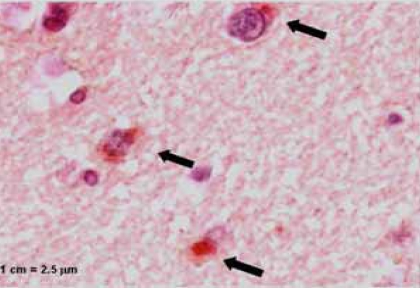
Immunohistochemical staining, showing the presence of *C. pneumoniae* (arrows) in ganglion cells of the brain. Magnification x400

**Fig. (3):**
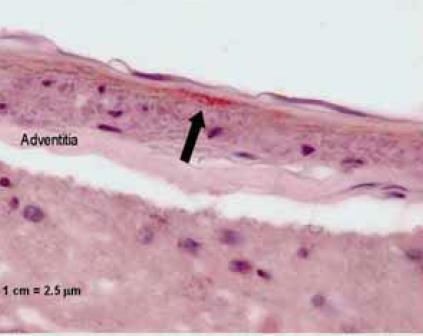
Immunohistochemical staining, showing the presence of *C. pneumoniae* (arrow) underneath the basal membrane, probably in myo-fibrocytes of the medial lamina of the arteriole. Magnification x 400

In 15/19 cases cerebral small vessel samples and brain tissue were available for neuropathological examination. SVD was present in either the basal ganglia or periventricular white matter in 13 of these. SVD was mostly observed in the basal ganglia, with some degree of SVD (changes to the cerebral small vessels or lacunar infarction) present in 9/15 cases. Signs of SVD in the periventricular white matter were found in 5/15 cases. When SVD was present, it was graded as mild, with concentric vessel wallthickening in small arteries and arterioles but with mild or minimal luminal narrowing, in 71 percent of the samples. Lacunar infarcts were found in 7/15 basal ganglia, and in 2/15 of periventricular white matter samples. We found only one case positive for CMV, and one for **C. pneumoniae*.* In the first case, which had died of basilar artery thrombosis, CMV was found in the basal ganglia. In the second case, which died from a post-operative bleeding after a laparotomy, *C. pneumoniae* was found in the periventricular white matter (Fig. **1**). IHC showed the presence of *C. pneumoniae* in the PCR positive *C. pneumoniae* was present in ganglion cells (Fig. **2**) and underneath the basal membrane, probably in myo-fibrocytes of the medial lamina of the arteriole (Fig. **3**). By IHC the presence of CMV in the PCR positive sample could not be confirmed. Since PCR did not show any HSV-1 DNA in any of the samples, IHC for HSV-1 was not performed.

### Serology

IgG antibodies against *C. pneumoniae* were found in 15/19 cases, IgG antibodies against HSV-1 in 16 and, IgG antibodies against CMV in 18.

Both cases that were found positive in PCR had antibodies against CMV and *C. pneumoniae*.

## DISCUSSION

### Cerebral Large Vessel Disease

We failed to establish the presence of *C. pneumoniae*, CMV or HSV-1 DNA in large vessel samples of patients who died from various causes, but who all had signs of atherosclerosis in at least one of the cerebral arteries, varying from intima fibrosis to fully developed atherosclerotic plaques with calcifications.

The presence of microorganisms in atherosclerotic plaques is patchy. Therefore, detection rates vary between studies [25]. Even if the vessels are embedded and processed longitudinally, and are of considerable length like in our study, not all the material is used in the PCR. Therefore, although the PCR can detect up to one copy of DNA, false negative results may occur as a result of “sampling error”. This patchy presence of microorganisms is also responsible for the differences in results between IHC and PCR [26]. In one study, that used IHC to detect *C. pneumoniae* in the cerebral vessels of asymptomatic patients, *C. pneumoniae* was found in only two percent, even though these specimens showed various signs of atherosclerosis of the MCA [18].

When PCR was used to detect *C. pneumoniae* in fully developed atherosclerotic lesions, the microorganism was found in one third of the vessels [20]. For PCR, frozen sections as well as formalin fixed sections are used. Deparaffinisation of formalin fixed sections, which is needed to allow isolation of DNA from the material, might result in loss of DNA. However, Gass *et al. *showed that correctly processed material even when embedded in paraffin should provide enough DNA to establish the presence of the microorganism, even in samples that have been stored for years [22]. Since we used the same technique to process the material and extract the DNA, and found no inhibition, we assume that our technique is sufficient enough to detect the three microorganisms in formalin fixed brain tissue. Our PCR’s are among the most sensitive, with *C. pneumoniae* being detected from up to one copy of DNA and both CMV and HSV-1 from up to 10 copies of DNA. The presence of CMV DNA in atherosclerotic tissue was first reported in a study using formalin fixed sections; CMV was found in 94 percent of the samples [27]. Studies on the presence of CMV in atherosclerotic tissue showed a prevalence varying from 0% [28] to 94% [27]. Using frozen sections or paraffin embedded tissue for PCR analysis does not influence the prevalence of CMV. Therefore, it is unlikely that the absence of microorganisms in our samples is due to the fact that we used formalin fixed sections.

One limitation of our study is that we did not compare the presence of *C. pneumoniae*, CMV and HSV-1 DNA in other arterial sites in the human body. In one study, that compared the presence of *C. pneumoniae* in various arterial sites, significant differences were found in chlamydial presence, with the highest rate in the abdominal aorta (67%), and much lower rates in the and cerebral (2%) arteries. Several studies have shown a higher prevalence of *C. pneumoniae* DNA in carotid arteries compared to intracranial arteries, with prevalence varying from 15% to 83%[29-32]. CMV was found in 36% of atherosclerotic carotid arteries, HSV-1 in 11% [13]. Even though our results do not support the hypothesis that *C. pneumoniae*, CMV and HSV-1 play a conditional role in the generation of cerebral atherosclerosis, we cannot rule out that these microorganisms do play a role in clinically manifest large vessel disease, since the majority of our patient group was asymptomatic. Furthermore, these microorganisms do seem to play a role in the initiation of carotid atherosclerosis, which is a risk factor for clinical stroke.

### Cerebral Small Vessel Disease

We detected *C. pneumoniae* DNA in brain tissue in one out of 19 cases, and found CMV DNA in one other case. We could not detect HSV-1 DNA in any of the samples. PCR is considered the gold standard for detection of microorganisms, but it has several disadvantages. Since PCR techniques become more and more sensitive, even very small amounts of DNA can be detected, leaving the question open for the clinical relevance of some findings. Our PCR’s are among the most sensitive, with *C. pneumoniae* being detected from up to one copy of DNA and both CMV and HSV-1 from up to 10 copies of DNA. Another disadvantage is that PCR does not localize the microorganism within the tissue. In an attempt to localize the microorganisms we used immunohistochemistry on the samples positive in PCR, *C. pneumoniae* was found in the basal ganglia, more specifically in ganglion cells and underneath the basal membrane of a small arteriole. The CMV presence could not be confirmed by IHC.

Studies on the prevalence of *C. pneumoniae*, CMV and HSV-1 in brain tissue, detected these microorganisms in 95 [33], 94 [34] and 67 [35] percent. However, the brain samples tested in these studies came from the frontal and temporal cortex, whereas all the patients had clinically manifest dementia. No studies have been reported on the presence of *C. pneumoniae*, CMV or HSV-1 in the basal ganglia or the periventricular white matter, the areas of the brain supplied by small vessels.

Our results do not point at an important role of the microorganisms in the generation of SVD. On the other hand, our results cannot deny any role of these microorganisms in eventual sustenance or acceleration of evident small vessel disease, which can only be accomplished with studying cases with clinically manifest small vessel cerebrovascular disease. However, with the early case fatality rate in small vessel stroke being low, such study would not be easy to perform. Although signs of small vessel disease were found in 13 of our 15 cases, none had clinically manifest disease.

In contrast to our findings with PCR and IHC, antibodies against *C. pneumoniae*, CMV and HSV-1 were found in the majority of the cases. This concurs with the prevalence of these antibodies in a general population. Positive antibody titres do not predict the presence of microorganisms in the vascular wall of cases, whereas the opposite is also true [36].

## CONCLUSIONS

We were unable to detect *C. pneumoniae*, CMV or HSV-1 in atherosclerotic large cerebral vessels, despite the presence of moderate to severe atherosclerotic changes in these vessels. Except for one case, in which *C. pneumoniae* and CMV were found in a sample of basal ganglia and periventricular white matter, this was also the case with respect to the cerebral small vessels. Our findings suggest a limited role for *C. pneumoniae*, CMV and HSV-1 in large and small vessel atherosclerosis. Whether these microorganisms play a significant role in symptomatic cerebral LVD and different types of SVD needs to be determined in further studies.

## Figures and Tables

**Table 1.** Presence and Severity of SVD and LVD, Age, Sex and Cause of Death in the 19 Studied Cases. F: Female, M: Male, NA: Not Assessed

Case	Sex	Age	LVD	SVD	Cause of death
1	F	80.0	+	NA	Hemorrhagic stroke
2	M	51.0	0	NA	Hepatic encephalopathy
3	M	87.0	++	+	Ischaemic stroke
4	M	73.0	+++	NA	Cerebellar haematoma
5	F	57.0	+	NA	Gastrointestinal bleeding
6	M	74.0	++	+	Cardiac arrest
7	M	62.0	+	+	Respiratory arrest
8	M	63.0	+++	+++	Hemorrhagic stroke
9	M	47.0	+	++	Cerebral metastasis
10	M	39.0	+	0	Sepsis
11	M	81.0	+++	+	Ischaemic stroke
12	F	70.0	++	+	Non-Hodgkin lymphoma
13	F	33.0	+	+	Myocardial infarction
14	M	79.0	+++	++	Hemorrhagic stroke
15	F	86.0	+++	+	Post op bleeding
16	M	69.0	++	+++	Myocardial ischaemia
17	F	86.0	+++	+	Meningitis
18	M	65.0	+	+	Myocardial ischaemia
19	M	79.0	++	0	Aortic dissection
